# Associations of smartphone usage patterns with sleep and mental health symptoms in a clinical cohort receiving virtual behavioral medicine care: a retrospective study

**DOI:** 10.1093/sleepadvances/zpad027

**Published:** 2023-07-05

**Authors:** Jonathan Knights, Jacob Shen, Vincent Mysliwiec, Holly DuBois

**Affiliations:** At time of submission: Mindstrong Health, Menlo Park, CA, USA; At time of submission: Mindstrong Health, Menlo Park, CA, USA; Department of Psychiatry and Behavioral Sciences, University of Texas Health Science Center at San Antonio, San Antonio, TX, USA; At time of submission: Mindstrong Health, Menlo Park, CA, USA

**Keywords:** smartphone, passive sensing, sleep disturbances, anxiety, depression, mental health disorders, sleep regularity, mhealth

## Abstract

**Study Objectives:**

We sought to develop behavioral sleep measures from passively sensed human-smartphone interactions and retrospectively evaluate their associations with sleep disturbance, anxiety, and depressive symptoms in a large cohort of real-world patients receiving virtual behavioral medicine care.

**Methods:**

Behavioral sleep measures from smartphone data were developed: daily longest period of smartphone inactivity (inferred sleep period [ISP]); 30-day expected period of inactivity (expected sleep period [ESP]); regularity of the daily ISP compared to the ESP (overlap percentage); and smartphone usage during inferred sleep (disruptions, wakefulness during sleep period). These measures were compared to symptoms of sleep disturbance, anxiety, and depression using linear mixed-effects modeling. More than 2300 patients receiving standard-of-care virtual mental healthcare across more than 111 000 days were retrospectively analyzed.

**Results:**

Mean ESP duration was 8.4 h (*SD* = 2.3), overlap percentage 75% (*SD* = 18%) and disrupted time windows 4.85 (*SD* = 3). There were significant associations between overlap percentage (*p* < 0.001) and disruptions (*p* < 0.001) with sleep disturbance symptoms after accounting for demographics. Overlap percentage and disruptions were similarly associated with anxiety and depression symptoms (all *p* < 0.001).

**Conclusions:**

Smartphone behavioral measures appear useful to longitudinally monitor sleep and benchmark depressive and anxiety symptoms in patients receiving virtual behavioral medicine care. Patterns consistent with better sleep practices (i.e. greater regularity of ISP, fewer disruptions) were associated with lower levels of reported sleep disturbances, anxiety, and depression.

**Statement of Significance**

Smartphone usage proximal to sleep is considered a risk factor for sleep disturbances and mental health symptoms. Currently, there is a lack of evidence for the associations between smartphone-based measurements and clinical symptoms in patients. From a large cohort of patients receiving virtual behavioral medicine care, we provide evidence that supports the utility of human-smartphone interaction behavior as an unobtrusive monitoring tool for symptoms that are highly prevalent in both clinical and nonclinical populations, specifically sleep disturbances, anxiety, and depression. This provides a basis for further study and refinement of the associations between human-smartphone behaviors and their timing with self-reported symptoms to integrate this model into clinical practice.

## INTRODUCTION

Smartphone usage is ubiquitous throughout society with 85% of adults owning a smartphone [1]. However, there is some variation in ownership, with 95% of adults younger than 49 years having a smartphone compared to 61% in adults greater than 65 years of age [2]. These devices, through their multiple sensors, can provide insight into an individual’s health-related behaviors [3, 4], circadian rhythms, and sleep-related outcomes [5, 6]; however, the current evidence exploring the negative impacts on sleep and mental health from smartphone usage during and around periods of sleep does not include large real-world clinical cohorts followed over long durations.

While there have been a growing number of studies, especially in the mental health and physical activity domains, these studies have been performed in small samples (often fewer than 50 participants who are typically not clinical patients) for relatively short, or unspecified, durations of data collection [7]. In one review which specifically focused on smartphone-based sleep assessments in clinical mental health cohorts, Aledavood et al. [8] evaluated tracking of sleep in patients with depression, anxiety, and psychotic disorders: Only eight studies were identified that evaluated mobile sleep sensing in serious mental illness (SMI), with sample sizes ranging from 7 to 61 patients and all studies lasting less than 12 months.

In contrast to clinical populations, the temporal pattern of smartphone usage behavior in nonclinical populations has been relatively well established to correlate with standard circadian patterns [9–11]—for example usage increasing in the morning, peaking in the early evening, and subsequently decreasing during the nighttime—and was initially shown capable of inferring relevant aspects of sleep, such as sleep duration and circadian preference in [12]. A larger subsequent study demonstrated that sleep measures from standard wrist-worn actigraphy, including sleep onset and offset correlated well with smartphone tappigraphy (i.e. touchscreen interactions while the device is in the unlocked state) [13]; however, sleep duration measured by smartphone usage was shorter than actigraphy in the majority of participants, with ~80% having smartphone usage—consistent with wakefulness—during their actigraphy detected sleep periods. These studies provide a basis that periods of smartphone inactivity follow a circadian pattern and correlate with sleep onset, offset, and duration, and that smartphone usage during the inferred sleep period (ISP) may be frequent and indicative of sleep fragmentation or disruptions.

The importance of sleep health, which includes sleep duration, timing or regularity, and efficiency, for both physical and mental wellbeing, is increasingly recognized throughout society [13]. While smartphone applications can evaluate sleep and circadian rhythms [14], and act as a screening tool for some sleep disorders [15], smartphone usage prior to, and during, the sleep period can result in sleep disturbances including difficulties falling asleep, shortened sleep duration and daytime impairment [16]. A majority of the evidence on the impacts of smartphone usage in and around bedtime has been performed in children and adolescents: in a meta-analysis of 20 studies including 125 198 US children with a mean age of 14.5 ± 2.2 years, phone usage pre-bedtime significantly increased the likelihood of having insufficient sleep duration and daytime sleepiness [17]. Studies in adults are more limited: the emerging evidence, though, supports nocturnal smartphone use as being associated with worse overall sleep quality, daytime impairment, and fatigue [18], and that 2–3 nights or more of smartphone use can have a significant impact on daytime sleepiness, fatigue, and depressive symptoms when compared with no nocturnal smartphone use. The observation that sleep disturbances associated with nocturnal smartphone usage are associated with mental health symptoms is consistent with the growing recognition that sleep disturbances are a recognized risk factor for, and marker of, mental health disorders including anxiety and depression [19–21].

The outlined body of evidence suggests that evaluating smartphone usage patterns for regularity of inactivity, duration of inactivity, and usage during periods of sleep, is an increasingly important area of study to provide insight into sleep-related behavioral patterns. Further evidence has been compiled to support that passively assessed, objective smartphone measures, correlate with self-reported mental health symptoms and can be used to evaluate movement and mobility in nonclinical populations [7, 22]. However, this evidence arises from studies with small sample sizes (< 50 participants) across relatively short periods of time (i.e. < 3 months), which may not necessarily apply to real-world clinical populations. While these pilot studies demonstrate feasibility and acceptability of smartphone-based assessments in patients with SMI, to the best of our knowledge, the use of passive smartphone assessments to longitudinally evaluate both sleep and mental health symptoms in a large clinical cohort—including SMI—receiving treatment has not been reported.

In order to further expand and build upon the existing literature, we performed a retrospective analysis on existing passively collected smartphone data and self-reported clinical outcomes from a large clinical cohort of adult patients receiving virtual behavioral medicine care via their smartphones. We developed a framework for identifying periods of inferred sleep at the individual level and subsequently assessed the relationship between smartphone behavioral patterns within (and around) this period and multiple patient-reported clinical measures including sleep disturbances as well as symptoms of anxiety and depression. Our primary hypothesis was that lower regularity of, or greater disruptions during, the expected sleep period (ESP) would be associated with higher levels of reported sleep disturbances: The secondary hypotheses were that these behaviors would be associated with higher levels of depressive and anxiety symptoms.

## METHODS

### Participants

**Figure 1.**
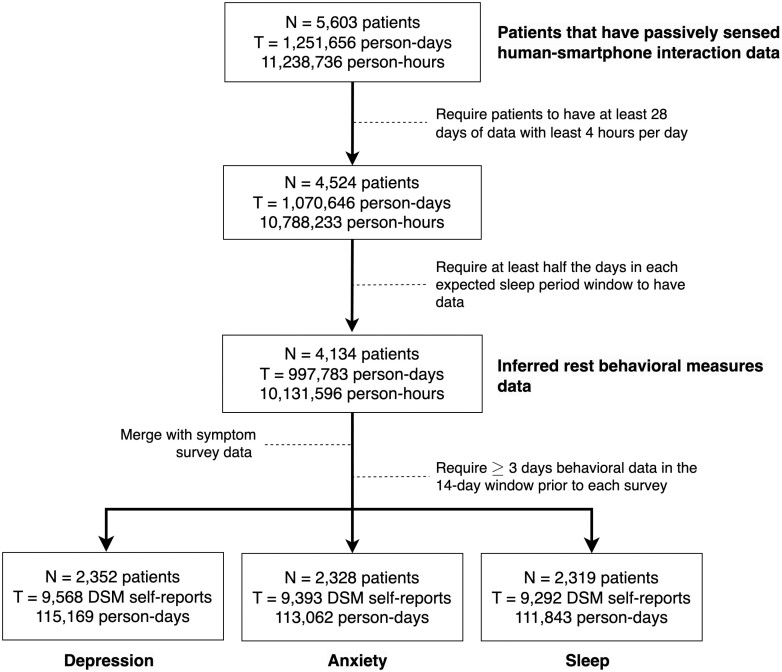
Flowchart of sample size (person, person-days, and self-reports) across the data processing steps.

In this retrospective study, patients who received standard-of-care virtual mental healthcare facilitated through the HEALTH mobile application (Mindstrong, Inc., Menlo Park, CA) between November 2020 and June 2022 were considered. Patients engaged with this system had previously been diagnosed with behavioral health conditions and referred to the sponsor through partnered healthcare plans, where licensed clinicians subsequently evaluated clinical status, including diagnoses. The inclusion requirements for the analysis sample were exclusively related to data properties, requiring at least 4 weeks of adequate data (described below in the *Data Processing and Operational Procedures* section and in Figure 1) and at least one applicable symptom severity measure. These requirements were defined as the minimum necessary data to conduct subsequent modeling between behavioral measures and clinical outcome reports (see cohort flow chart in Figure 1). Patient demographics are provided in Table 1 and summarized here: patient ages in the analysis sample ranged from 19 to 78 years old, with a mean of 56.7 (*SD* ± 11.3) years; 73.6% of the cohort identified as female, 25.1% as male and 0.3% did not specify their gender identity; race/ethnicity consisted of 48.9% white, 10.5% African American, 3.4% Hispanic/Latino, 2.1% multiracial, and 34.3% of the cohort did not specify their race/ethnicity; The most prevalent primary behavioral medicine diagnoses among patients in the analysis sample were major depression (40.6%), bipolar disorder (20.0%), and smaller samples of schizophrenia/schizoaffective disorder (6.5%), and personality disorder (3.5%). Of note, 55.3% of the patients in this sample resided in a rural setting.

**Table 1.** Descriptives of Demographics.

Descriptive	
Sample size, count	2352
Age, mean (SD)	56.7 (11.3)
Younger than 20 years old	< 0.01%
20-29 years old	1.8%
30-39 years old	7.1%
40-49 years old	15.6%
50-59 years old	28.4%
60-69 years old	35.0%
70-79 years old	11.7%
**Identified gender, percentage**	
Female	73.6%
Male	25.1%
Unspecified	1.0%
**Ethnicity, percentage**	
White	48.9%
Black or African American	10.5%
Hispanic/Latino	3.4%
Multiracial	2.1%
Other	0.9%
Unspecified	34.3%
**Primary diagnosis**	
Major depression	955 (40.6%)
Bipolar	471 (20%)
Personality disorder	81 (3.4%)
Schizophrenia (or Schizoaffective)	152 (6.5%)
Other	685 (29.1%)
Missing	8 (0.3%)
**Number of days regarding smartphone behavior data, mean (SD)**	310 (196.8)
Younger than 20 years old	NA
20-29 years old	270.5 (174.7)
30-39 years old	317.7 (224.4)
40-49 years old	337.5 (205.8)
50-59 years old	319.8 (198.6)
60-69 years old	297.7 (189.5)
70-79 years old	287.5 (181.1)
**Location**	
Urban	44.7%
Rural	55.3%

The average number of days of smartphone behavior data per patient was 310 (*SD* = 196.8, min = 15, max = 1088). Among these patients, 1586 had more than 180 days of smartphone behavior data. The quartile cutoffs for the empirical distribution of smartphone data (days of data) were 147, 285, and 447 days, respectively. There were differences in the availability of data across age groups, which was mitigated through inclusion of these demographic factors in the regression models (for more details, see Table 1 and Supplementary Report Part 2). The observation window for each individual was defined only by their duration—and extent—of engagement with virtual behavioral health care from the sponsor.

### Data processing and operational procedures

As part of routine clinical care, patients were asked to report their mental health symptoms at regular intervals (60 days prior to September 2021; 30 days after September 2021). Mental health symptoms were self-reported using the DSM-5 Self-Rated Level 1 Cross-Cutting Symptom Measure—Adult survey (DSM-5 L1) [23] on the HEALTH mobile application.

Smartphone keyboard and app usage metadata was collected unobtrusively on the Android operating system. The metadata includes various de-identified human-smartphone interactions (e.g. typing, scrolling, app change). The starting time of each event was recorded with a timestamp as granular as a millisecond. There were 5603 patients that had passively sensed human-smartphone interaction data and 1.25 million person-days throughout the observation period (see Figure 1 for details). To ensure sufficient smartphone usage data was present for analysis, there were two requirements for patient data: First, we required at least 4 unique hours of passive smartphone activity in a minimum of 28 days (4 weeks) across patients; and second, for each of the ESP intervals, which consisted of 30 days, at least half of the days (i.e. 15 days) were required to have adequate smartphone usage data in order to generate behavioral measures for that interval. After applying these criteria, the final sample for behavioral measures data was 4134 patients and approximately 997 000 person days (Figure 1). After subsequently merging the human-smartphone interaction data with the self-reports from the DSM-5 L1 data, there were between 2319 and 2352 patients across the three symptom domains of interest. The slight differences in sample sizes arise as there was no criteria that required patients to finish entire surveys on the HEALTH app, meaning each survey may have contained responses for all, some, or none of the desired domains.

Informed consent to use the app and have clinical and passively collected smartphone data used for research purposes was obtained from all patients before being enrolled into clinical care. This retrospective study was conducted under a secondary data analysis protocol to identify clinically relevant associations in active and passive data collection, which has been reviewed and approved as exempt by the WCG Institutional Review Board (formerly Western Institutional Review Board).

### Clinical measures

The clinical measures used in this study encompassed the domains of sleep disturbance, depression, and anxiety. These domains are part of the American Psychiatric Association’s DSM-5 L1 which was developed to evaluate initial symptoms and monitor treatment response across domains [24]. This questionnaire uses self-reported responses on a five-point Likert scale (0 = not at all/none, 1 = rare, less than a day or two/slight; 2 = several days/mild, 3 = more than half the days/moderate; 4 = nearly every day/severe) to evaluate how much or how often an individual has been bothered by a symptom during the last 2 weeks. The DSM-5 L1 questions can be used serially over regular intervals to track changes in symptoms over time. This questionnaire has been found to be clinically useful with test-retest reliabilities that are good to excellent [24]. For the purposes of this study, the following questions were used:

#### Sleep disturbance.

The symptom of sleep disturbance is assessed by one item on the DSM-5 L1. This question contains the prompt “during the past 2 weeks, how much (or how often) have you been bothered by problems with sleep that affected your sleep quality over all?”

#### Depressive symptoms.

Depressive symptoms are assessed by two items on the DSM-5 L1. Both items contain the prompt “during the past 2 weeks.” Item 1 measures anhedonia, which prompts “how often have you had little interest or pleasure in doing things?", item 2 measures depressed mood, which prompts “how often have you been feeling down, depressed, or hopeless?” We utilized the average of these two items as one depressive symptom score for the outcome variable in this analysis.

#### Anxiety symptoms.

Anxiety symptoms are assessed by three items on the DSM-5 L1. All items utilized the prompt “during the past 2 weeks.” Item 1 prompts “how often have you been bothered by feeling nervous, anxious, frightened, worried, or on edge”, item 2 prompts “how often have you been bothered by feeling panic or being frightened?”, item 3 prompts “how often have you been bothered by avoiding situations that make you anxious.” We averaged these three items as one anxiety symptom score for the outcome variable in this analysis.

### Smartphone behavioral measures

#### Pre-processing of smartphone data.

An individual’s interactions with their smartphone were collected unobtrusively in real-time and then securely transmitted and stored in HIPAA-compliant servers. Such interactions with the smartphone include: changing foreground applications, clicking, scrolling, typing, and changing views within an application (e.g. selecting a contact to message from a list of contacts), and turning the smartphone screen on. Phone activity was aggregated into 15-min bins to represent whether the person had interacted with the phone or not during that interval. Previous research has demonstrated that 15-min bins of screen on/off activity used to analyze inferred sleep were 89% accurate compared to sleep measured by consumer wearable technology [25]. The 15-min bins that have any human-smartphone interaction are considered active periods, and the 15-min bins that have no human-smartphone interaction are considered inactive periods. In sum, each day of a person’s phone activity is represented by a sequence of 96 active or inactive values (1 for active or 0 for inactive) corresponding to the ninety-six 15-min bins which fully encompass the 24-h period.

#### Longest period of smartphone inactivity.

Defined as the longest consecutive period where there was no human-smartphone interaction and can be used to determine an inferred sleep period (ISP) [12, 26]. We computed sleep in two ways in this analysis—a daily approach for the ISP, and a 30-day windowed approach for the expected sleep period (ESP).

#### Inferred sleep period.

The daily ISP constitutes the longest consecutive stretch of inactive bins in each day. Specifically, the ISP was computed by applying the run-length encoding (RLE) function in R (version 4.2.0) [27] to the 96-value sequence of daily smartphone activity from 00:00 am to 11:59 pm of the same day. The RLE function returns a list of consecutive active/inactive periods, and each consecutive active/inactive period in the list is represented with the starting bin index and the duration of this specific period. The ISP is selected as the inactive period of the longest duration in each day.

#### Expected sleep period.

**Figure 2.**
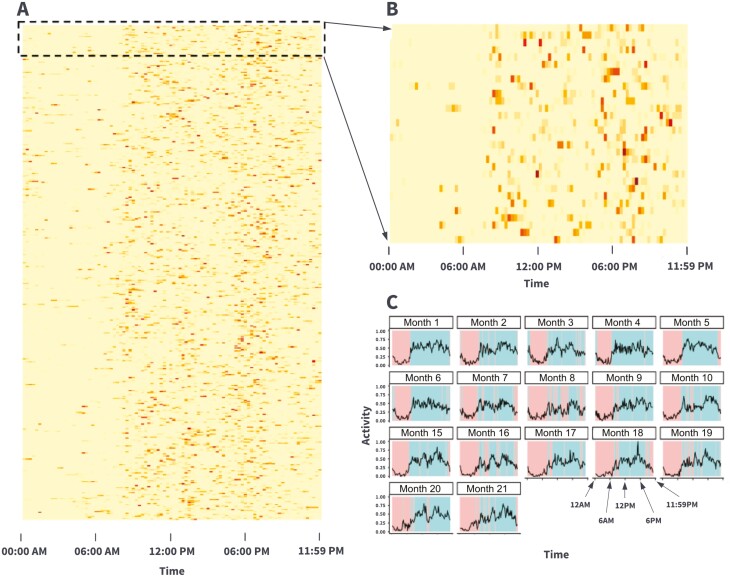
Illustration of the 30-day window approach. Panel A is a heatmap of one person’s human-smartphone interaction data over 499 days. Each row is a day, and each column is a 15-min bin from midnight of day *N* to midnight of day *N* + 1. The bins are colored from yellow to orange to red. Bins colored yellow indicate no smartphone activity, orange are some activity while bins colored red constitute the highest activity. Panel B is zooming into the first 30 days of the phone activity heatmap. Panel C is the binarized phone activity over the 30-day window per 15-min bin. Each smaller panel has the label of the starting date of the 30-day window. The *x*-axis is time, from midnight to midnight of the next day, and the *y*-axis is the percentage of phone activity. The background is colored as pink for periods classified as inactive or blue as active. Using the heatmap (A–B) and binarized phone activity (C) one can observe phone behavior usage shifting during the active periods from periods of consistent high activity (Month 1) to periods of relatively low phone use (e.g. Month 8) leading to more identified periods of inactivity. Figure 3C also highlights the personalized nature of the ESP, which allows this approach to change expectations and adapt if user behavior shifts on a monthly scale.

The 30-day windowed approach for the ESP represents a consistent pattern of low smartphone inactivity over a longer period, and thereby a more stable representation of expected behavior. The 30-day windowed approach consists of three steps. First, for each 15-min bin the percentage of days with activity in a consecutive 30-day window (no overlap between windows) was identified as the activity percentage (Figure 2A and B highlights the raw activity for an individual across time bins, and the 30-day aggregate fraction, respectively). Second, the percentage of activity was converted to a binary activity variable to allow for computing the ESP: K-means clustering was utilized to identify the person-level distribution of the percentage of activity into two clusters, with the cluster having a lower percentage centroid being considered the inactive data (binarized to 0), otherwise, the cluster was considered active (binarized to 1). Finally, with the binarized 96-value sequence (as shown in Figure 2C) for each 30-day window, we computed the ESP in the same fashion as the computation of the daily ISP.

#### Overlap percentage.

**Figure 3.**
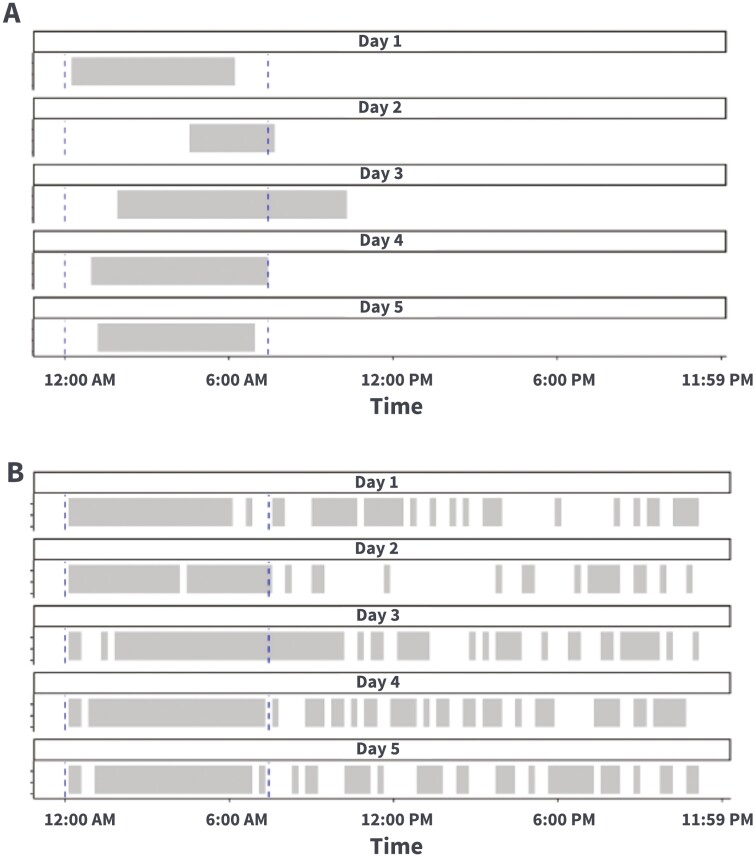
Illustration of daily overlap percentage and daily disruption. Panel A shows the daily overlap percentage for 5 days. The gray bar is the daily ISP and the blue dashed line is the 30-day windowed ESP. The 30-day windowed ESP starts at 00:00 am and ends at 07:45 am. Panel B shows daily disruptions within the 30-day windowed ESP for the same corresponding 5 days. The gray bar indicates smartphone inactivity, and the white bar indicates smartphone activity. ISP = inferred sleep period; ESP = expected sleep period.

The overlap percentage of the ESP is defined as the proportion of the ISP on each day that falls within the ESP. See Figure 3a for a visualization of the daily ISP (gray bar) overlapping with the ESP (blue dashed line starting at bin index 96, now moved to index 0 to illustrate the window, and ending at bin index 31). The number of overlapping bins with the ESP for these 5 days are 25, 12, 27, 23, and 24, respectively (percentage: 100%, 92%, 66%, 100%, and 100% of the daily ISP). The intent of the overlap percentage was to quantify the regularity of the daily period of smartphone nonusage as a marker approximating sleep onset and offset, not the regularity of the duration of smartphone nonusage periods. Thus, this calculation allowed periods of nonusage, regardless of duration, that were within the 30-day windowed ESP to have an overlap percentage of 100%.

#### Disruption.

Disruption, or presumed periods of wakefulness, is defined as the total smartphone interactions in each day which occur during the corresponding 30-day ESP. To characterize disruption, the number of 15-min bins with activity during the ESP are counted. See Figure 3b for visualization of 5 days’ disruption (white space indicates activity, gray indicates inactivity) within the ESP (marked by a blue dashed line starting at 00:00 am, and ending at 07:45 am). The sum of 15-min bins with disruptions for these 5 days are 5, 2, 4, 3, and 4 disruptions, respectively.

### Analysis procedures

Given the nested nature of repeated measures longitudinal data, hypotheses were examined within a hierarchical modeling framework. Following the recommended practice [28], the predictor variables were split into time-invariant (between-person differences) and time-varying (within-person changes) components. We evaluated associations between the inferred sleep behavioral measures (ESP and ISP) and three outcome variables, namely sleep disturbances, depressive symptoms, and anxiety symptoms. Thus, three independent models were run where the first model used sleep disturbances as the dependent variable, the second model used depressive symptoms as the dependent variable, and the third model used anxiety symptoms as the dependent variable, while keeping the predictors the same. Behavioral measures were aggregated to average values for the fourteen days prior to each survey response—only intervals that had at least 3 days of viable behavioral data were included in the analysis.

In order to control for potential confounding impacts from demographics and clinical profiles, all demographics listed in Table 1—with the exception of ethnicity (due to high missingness)—were independently regressed against the three symptom report variables to identify significant relationships (see Supplementary Report Section 1). All variables that were significant with at least one outcome variable (*p* < 0.05) were included in the three separate hierarchical models. The significant demographic variables included were: age, identified gender, primary behavioral medicine diagnoses of major depression, personality disorder, or schizophrenia, and urban/rural location.

Relationships among the extended set of variables were then modeled using two-level models of the following form:


$$\begin{array}{l} \left\{{\text{SleepDisturbance}}_{it},\;{\text{Depressive}}_{it},{\text{Anxiety}}_{it}\right\}=\beta_{oi}+\beta_{1i}\times \text{wp}{\text{.OverlapPercentage}}_{it}+\beta_{2i}\times \\ \text{wp}{\text{.SleepDisruption}}_{it}+\beta_{3i}\times \text{wp}{\text{.Duration}}_{it}+e_{it} \end{array}$$ (1)

$$\begin{array}{l} \beta_{0i}=\gamma_{00}+\gamma_{01}\times \text{bp}{\text{.OverlapPercentage}}_{i}+\gamma_{02}\times \text{bp}{\text{.SleepDisruption}}_{i}+\\ \gamma_{03}\times \text{bp}{\text{.Duration}}_{i}+\gamma_{04}\times {\text{Age}}_{i}+\gamma_{05}\times {\text{Gender}}_{i}+\gamma_{06}\times \text{Y }\_{\text{ MajorDep}}_{i}+\\ \gamma_{07}\times \text{Y }\_\;{\text{ PersonalityDisorder}}_{i}+\gamma_{08}\times \text{Y }\_\;{\text{ Schizophrenia}}_{i}+\gamma_{09}\times {\text{Location}}_{i}+u_{0i} \end{array}$$ (2)

\begin{eqnarray*} {{\beta }_{1i}}={{\gamma }_{10}}+{{u}_{1i}} \end{eqnarray*} (3)

\begin{eqnarray*} {{\beta }_{2i}}={{\gamma }_{20}}+{{u}_{2i}} \end{eqnarray*} (4)

\begin{eqnarray*} {{\beta }_{3i}}={{\gamma }_{30}}+{{u}_{3i}}\end{eqnarray*} (5)

where the repeated measures of sleep disturbances, depressive, or anxiety symptoms for individual *i* on survey *t* are modeled as a function of person-specific intercepts ($ {{\beta }_{0i}} $) that indicate baseline level of the outcome variable, and the “ $ Y\_$ *” nomenclature represents a binary variable equal to 1 if individual *i* has that primary diagnosis and 0 if not. The person-specific coefficients ($ {{\beta }_{\left\{1-3 \right\}i}} $) indicate the extent of within-person associations between behavioral measures and the outcome variable of interest. $ {{\gamma }_{00}} $ to $ {{\gamma }_{30}} $ are the sample-level parameters. $ {{u}_{0i}} $ to $ {{u}_{3i}} $ are the residuals of unexplained between-person differences and are assumed multivariate normal with mean equal to zero and variances $ \sigma _{{{u}_{0i}}}^{2} $, $ \sigma _{{{u}_{1i}}}^{2} $, …, $ \sigma _{{{u}_{3i}}}^{2} $.

The models were fit to the data using the *lme4* package in R (version 1.1-29) [29] with incomplete data (3.5%) treated as missing at random. To avoid undue influence of extreme values in the regression models, data points that were more than four times Cook’s distance [30] were removed prior to modeling. Cook’s distance was generated from an initial regression of the data using (1)–(2) with no demographic variables. The decision to leverage Cook’s distance and remove data points with disproportionate impacts on the behavioral regressions—as opposed to data modification techniques such as winsorization—was rooted in the intention to explicitly focus the definition of extreme values to the context of the defined linear model: given the lack of characterization in the literature of these novel behavioral measures, adopting data modification techniques to conform the data to the observed distributions may not be appropriate at this time. Data comparisons between the distributions of dependent and independent variables for the unadjusted and adjusted data sets were performed using the nonparametric Kolmogorov–Smirnoff test. Model diagnostics and goodness-of-fit plots are provided in Supplementary material, Supplementary S1 Part 4). Note that standard goodness-of-fit statistics are not presented in Table 2 for two reasons: (1) In the DSM-5 L1, all three of the presented symptom domains have different numbers of questions, and thereby different levels of detail/specificity representing their magnitude, indicating that cross-model comparisons of fits is not truly representative of relative performance; and (2) it is not the intention of this analysis to present the most appropriate model for the clinical domains under investigation—rather, the intention is to identify if significant relationships exist between the mental health symptoms of interest and the defined smartphone behavioral metrics after controlling for available relevant clinical and demographic factors.

**Table 2.** Results From the Multilevel Model Examining the Association Between Sleep Disturbance, Depressive and Anxiety Symptoms and Human-Smartphone Interaction Behavior

Parameters (unstandardized values)	Sleep disturbance		Depressive symptoms		Anxiety symptoms	
	Estimate	CI	Estimate	CI	Estimate	CI
**Fixed effects**						
Intercept (*γ*00)	2.49^***^	0.54	2.37^***^	0.44	2.51^***^	0.46
wp.OverlapPercentage (*γ*10)	−0.21^*^	0.40	0.015	0.31	−0.07	0.28
wp.Disruption (*γ*20)	0.045^***^	0.027	0.0004	0.021	0.013^**^	0.019
wp.Duration (*γ*30)	−0.03^**^	0.040	0.0055	0.032	−0.0078	0.028
bp.OverlapPercentage (*γ*01)	−0.90^***^	0.86	−0.80^***^	0.69	−0.66^***^	0.72
bp.Disruption (*γ*02)	0.098^***^	0.038	0.053^***^	0.031	0.048^***^	0.033
bp.Duration (*γ*03)	0.015	0.066	0.061^***^	0.053	0.069^***^	0.056
Age	−0.0044^†^	0.0089	−0.0034^†^	0.0072	−0.011^***^	0.0075
Gender^a^ (Female reference)	−0.0012	0.22	−0.020	0.18	0.077	0.18
Location (Urban reference)	−0.065	0.19	−0.065	0.15	−0.076^†^	0.16
PD major depression	−0.0052	0.20	0.086^*^	0.16	−0.078^†^	0.17
PD personality disorder	0.11	0.51	0.28^**^	0.41	0.21^†^	0.44
PD schizophrenia	−0.40^***^	0.40	−0.27^**^	0.32	−0.17^*^	0.34
Random effects						
Variance of Intercept ($ \sigma _{{{u}_{0i}}}^{2} $)	0.97		0.65		0.75	
Variance of random slope wp.OverlapPercentage ($ \sigma _{{{u}_{1i}}}^{2} $)	0.28		0.020		0.0000	
Variance of random slope wp.Disruption ($ \sigma _{{{e}_{it}}}^{2} $)	0.0012		0.0005		0.0008	
Variance of random slope wp.Duration ($ \\sigma _{{{u}_{3i}}}^{2} $)	0.0082		0.0033		0.0023	
Variance of residual ($ \sigma _{{{e}_{it}}}^{2} $)	0.67		0.43		0.35	

^a^= Patient’s identified gender; PD = Primary Diagnosis; CI = 95% confidence interval; wp = within-person portion of the predictor; bp = between-person portion of the predictor; ^†^*p* < 0.1, *p < 0.05, ** *p* < 0.01, ***p < 0.001.

Finally, we performed bootstrapping of the data to assess how robust the associations on the larger data set would be on smaller sample populations (in the same cohort). During this process the models were re-estimated on 500 random samples (with replacement) using 20% of the original population sampled at the ID level—all data for each selected ID was retained in each sample. These results are briefly discussed in the main body of the text with details provided in Supplementary Report Part 7.

## RESULTS

### Summary statistics of the measurements

Summary statistics of the behavioral measurements of human-smartphone interactions and symptom survey reports are described briefly here and provided in more detail in Supplementary Report Part 2.

The median observed completion rate within the individual observation windows (elapsed duration between first and last observed survey) for patients in this data set was 78% for depression and anxiety, and 75% for sleep disturbances. The 25th-percentile and 75th-percentile for all three outcomes were 50% and 100%, respectively. The data contained slight differences in the average symptom survey count available for each patient across the acuity spectrum; however, there was not a systematic trend observed across all symptoms, and the differences were only between 1 and 2 surveys. Further details can be found in Supplementary Material, Supplementary S1 Part 3.

The mean level of overlap percentage was 74% (*SD* = 18%), the mean number of time windows during the ESP with disruptions was 4.85 (*SD* = 3.03), and the mean duration of the ESP was 8.4 h (*SD* = 2.3). For symptom severity reports, the mean self-reported sleep disturbance level was 2.16 (*SD* = 1.37), the mean depressive symptom level was 2.15 (*SD* = 1.23), and the mean anxiety symptom level was 1.86 (*SD* = 1.36). All correlations between behavioral measures and clinical reports at the population level were negligible (none exceeded an absolute value 0.3, details provided in Supplementary Report Part 2).

### Associations between self-reported sleep disturbance and human-smartphone interaction behavior

For reported sleep disturbances, 5.99% of the observations fit the Cook’s distance criteria for removal. There were no changes in the distributions of the symptom severity reports or the behavioral distributions (see Supplementary S1 Part 3) following the data cleaning.

The degree of self-reported sleep disturbances on the DSM-5 L1 for a typical individual was 2.49 ($ {{\gamma }_{00}} $ = 2.49, *p* < 0.001) on a 0 to 4 scale (see Table 2). The within-person association between sleep disturbances and overlap percentage was significant ($ {{\gamma }_{10}} $ = −0.21, *p* < 0.05), meaning that when a participant’s overlap percentage was compared with their own average, higher values (greater regularity) were associated with lower degrees of self-reported sleep disturbances. The between-person association between sleep disturbance and overlap percentage was also significant ($ {{\gamma }_{01}} $ = −0.90, *p* < 0.001), indicating that participants with higher overlap percentages (greater regularity of smartphone inactive periods) had lower levels of self-reported sleep disturbance. The within-person association between sleep disturbances and disruption was also significant ($ {{\gamma }_{20}} $ = 0.045 *p* < 0.001): When comparing a participant’s daily disruption with their own average, higher values were associated with higher degrees of self-reported sleep disturbance. Furthermore, the between-person association between sleep disturbance and disruption was also significant ($ {{\gamma }_{02}} $ = 0.098, *p* < 0.001), meaning that participants with more disruptions during the ESP also reported higher levels of sleep disturbances. The within-person estimate of ESP duration was significant ($ {{\gamma }_{30}} $ = −0.03, *p* < 0.01): comparing a participant’s ESP duration with their own average, longer durations were associated with lower levels of self-reported sleep disturbance. Visualizations for relationships between self-reported sleep disturbances and behavioral measure are provided in the Supplementary Report Part 5.

For the within-person effects, disruptions had 88% agreement across sub-samples (detection power at 0.05 significance level) during the bootstrapping process indicating a strong and replicable significant association between degrees of reported sleep disturbance and the amount of disruptions observed from the data. Within-person effects of ESP duration and overlap percentage were less consistent at 22% and 12% detection power, respectively, indicating that these values likely impact subgroups of patients differently and would be hard to replicate on smaller data sets. For the between-person effects, disruption had perfect agreement across sub-samples, while the overlap percentage was significant in around 39% of the sample populations.

### Associations between depression or anxiety symptoms and human-smartphone interaction behavior

For reported depression and anxiety data, 5.89% and 5.7% of the observations fit the Cook’s distance criteria for removal, respectively. There were no changes in the distributions of the symptom severity reports or the behavioral distributions (see Supplementary S1 Part 3) from the data cleaning process.

For depressive symptoms, the between-person associations with overlap percentage, disruptions, and ESP duration were significant (see Table 2) and directionally consistent with the original hypotheses—patients with higher levels of regularity, as measured by the overlap percentage, reported lower levels of depressive symptoms, while patients with higher levels of disruptions during their ESP and/or longer ESP durations, reported higher levels of depressive symptoms. Regarding the stability of these relationships to sub-sampling on the data, disruption was identified as significant around 84% of the time, while overlap percentage and ESP duration were both detected roughly 47% of the time.

For anxiety symptoms, the between-person relationships for the behavioral measures and symptom severity were qualitatively similar, and directionally identical, to the depressive symptom reports (see Table 2); however, there were differences in the robustness of these relationships to be detected in the bootstrapped sub-populations. The between-person effects of disruptions and overlap percentage with anxiety symptom severity each had lower power to be detected on the bootstrapped samples: The impacts of disruptions and overlap percentage on anxiety symptoms were detected with around 68% and 32% power, respectively (as opposed to around 84% and 47%, respectively, for depression), while the impact of ESP duration was detected in around 56% of the bootstrapped samples (as opposed to 47% for depression). See Supplementary Report Part 7 for full details.

In addition to the between-person effects with anxiety symptoms, the within-person relationship with ESP disruptions was also significant ($ {{\gamma }_{20}} $ = 0.013, *p* < 0.01) but had low power to be detected across smaller bootstrapped samples—around 19% of bootstrapped populations identified significant relationships between anxiety symptom levels and within-person ESP disruptions.

## DISCUSSION

### A framework for passive smartphone usage patterns to infer sleep

The ability to leverage passively collected smartphone usage data to address sleep and mental health symptoms has long been postulated [31]. As a majority of smartphone users bring their devices to bed [32, 33], their usage patterns during this time period provide the ability to evaluate wakefulness and inferred sleep. Previous studies have evaluated small nonclinical populations using objective smartphone measurements (i.e. screen on/off [25], accelerometry [34], light sensing, passive usage [12], tappigraphy [13]) to determine inferred sleep. In order to measure sleep-related outcomes from passively collected smartphone data we developed a novel approach with a large clinical population to calculate and expand upon previously described measures, including the ESP, the daily longest period of inactivity (ISP), disruptions (daily phone usage during the ESP), and the overlap percentage (regularity of the ISP compared to the expected period of smartphone inactivity, ESP). The basis of our calculations for the overlap percentage and disruptions was the ESP—in this instance calculated using a 30-day historical window—which provided a more robust foundation for a habitual sleep pattern. While the period of inactivity does not definitively determine sleep, a similar smartphone-based assessment performed as well as, if not better than, actigraphy [13]. This was primarily due to the disruptions captured with active smartphone usage that definitively establish periods of wakefulness but were not captured by actigraphy and highlight that smartphone and wearable devices likely form complementary behavioral data sets with distinct and unique value.

### Sleep disturbances

Utilizing passively collected objective smartphone data to assess sleep disturbances in a large cohort of patients receiving virtual behavioral medicine care, our primary finding is that disturbances from smartphone usage (i.e. disruptions) during the ESP are associated with greater self-reported sleep disturbances. This finding was consistent at both an individual (within person) and a population (between person) level, and was additionally captured as significant across bootstrapped sub samples of the data. This provides ecologically valid objective findings that support previous studies which found self-reported nocturnal smartphone usage to be associated with poor quality sleep and sleep disturbances [15, 35]. Given the current view that sleep disturbances are considered a “predictive prodromal symptom” [36] of behavioral medicine disorders as opposed to the previous view of an epiphenomenon, the finding that smartphone usage patterns can potentially facilitate earlier diagnosis and treatment of sleep disturbances in patients with mental health disorders is important [37].

### Overlap percentage: a potential measure of sleep regularity

An evolving concept of sleep health is sleep regularity, or sleep patterns, whereby an individual has either regular sleep with a consistent onset and offset, or variable sleep with changes in the onset and offset of their sleep period [38–40]. It is postulated that regularity of sleep is likely as important as sleep duration, noting a regular sleep pattern is not only an important marker of good quality sleep, but also for physical and mental health, as well as performance [39, 41]. Historically, sleep regularity has been primarily evaluated with sleep diaries and actigraphy [42]. Given the regular and habitual use of smartphones, we utilized the overlap percentage as a potential way to assess sleep regularity on a longer-term basis. Overlap percentage, which measured how much a given day’s ISP overlapped with the ESP, is similar to measures of sleep regularity [35]: this is supported by the alignment between a mean overlap percentage of 75% in the present cohort and a median sleep regularity index score of 81% in a cohort of 60 997 study participants in the United Kingdom [40]. The lower overlap percentage in our cohort may be due to evaluating this in patients with SMI as opposed to volunteer participants. Our finding that a lower overlap percentage was associated with greater self-reported sleep disturbances and increased symptoms of anxiety and depression is consistent with the literature that variability in an individual’s sleep-wake pattern is detrimental to overall physical and mental health [40, 43].

Although the relationship between overlap percentage and reported sleep disturbances was moderately replicable across sub-populations, for example those patients with higher overlap percentages had lower levels of reported sleep disturbances, this behavioral measure was found to have low concordance across smaller sub-populations when comparing individuals to their own data. More accurately, our findings suggest that the importance of shifting measures of regularity with respect to sleep patterns at an individual level is likely context dependent. This is different from the findings of the impacts of disruptions during the ESP, which were robustly identified as significant for both changes within an individual, as well as across individuals.

One factor that may impact the sensitivity of the overlap percentage at an individual level is that the current implementation is naïve to the duration of that day’s ISP and the duration of the ESP. Together, the existence of a moderate correlation between overlap percentage and ESP duration (*r* = 0.6, see Supplementary Report Part 2), as well as the observation that the impact of within-person changes in ESP duration was more often found to be significant across bootstrapped sample populations (see Supplementary Report Part 7), suggest that including the duration of daily sleep for measures of regularity would be more beneficial from an analysis standpoint than keeping them separate. It is also possible that simple benchmarking of individual patient levels would inform whether or not changes in regularity measures would be expected to be impactful: For instance, in patients who have high levels of sleep regularity, intermittent deviations may be natural and not impactful to overall sleep quality, whereas for patients who have historically low levels of sleep regularity, improving this measure of sleep health may have large impacts.

### Mental health symptoms of depression and anxiety

In this analysis, increased disruptions and a lower overlap percentage were associated with greater symptoms of anxiety and depression. This is consistent with previous research in smaller clinical populations [8]. One such study evaluated passive smartphone usage in 47 patients with diabetes in which those with co-occurring depression had significantly greater usage from midnight to 06:00 than those without depression [44]. Conversely, in a study of 815 university students leveraging self-reported sleep periods over 16 weekdays, nocturnal smartphone usage was not strongly associated with increased depressive symptoms [45]. The findings in the current work—that increased disruptions to, and lower overlap percentages with, the ESP were associated with greater symptoms of depression and anxiety—suggests not only support for the concept that poor sleep quality in a clinical population (as opposed to healthy young adults) is associated with the exacerbation of mental health symptoms [46], but also that utilizing fixed windows of expected behavior (e.g. midnight to 06:00, or the ESP used here) when assessing the impacts of sleep disruptions on mental health symptoms may be a more sensitive approach than using shifting daily sleep periods by themselves.

While the underlying pathophysiology for the association with sleep and mental health is not fully understood, one study compared scheduled nocturnal awakenings to a similar duration of sleep restriction without awakenings to determine which aspect of sleep disturbances had a greater impact on mood [47]. Following sleep periods with nocturnal awakenings, participants had a significantly lower mood than those with shortened sleep periods; thus, it does not appear to be sleep duration per se, but awakening during sleep that likely contributes to depressive symptoms. This provides a potential basis for how disturbances or awakenings related to smartphone usage are associated with increased mental health symptoms.

While the duration of the ESP was significantly associated with symptoms of anxiety and depression—whereby a longer ESP was associated with increased anxiety and depressive symptoms—this finding is likely due to the nature of our clinical mental health cohort, where evidence from similar populations has demonstrated long sleep duration to be associated with depression, and has been found to be predictive of the persistence of symptoms of anxiety and depression [42, 43]. However, additional studies evaluating inferred sleep in patients with and without behavioral medicine disorders are required to further elucidate this finding.

### Strengths and limitations

The major strength of our study is that it was performed using objective smartphone data from a large cohort of clinical patients undergoing routine virtual mental healthcare. This differs from previous studies which utilized self-reported smartphone usage or were performed using objective measures in small or nonclinical cohorts for relatively short periods of time.

Despite the size of the data, this was a retrospective study utilizing observational data, which had limitations. First, only primary mental health diagnoses were included. This excluded the potential for robust clinical characterization of comorbidities and how those comorbidities may influence the identified associations—this is an important area of future studies. Furthermore, generalizability of these findings across the acuity spectrum requires further investigation, which is supported in the data through the identification of a subset of high acuity patients (with fewer symptom observations) that were removed from the analysis sample as disproportionately impactful to the regression models. Second, the ability to determine the directionality of the findings was not possible—specifically, if smartphone usage during the ISP contributed to the self-reported sleep disturbances or if sleep disturbances associated with sleep or behavioral medicine disorders resulted in greater smartphone usage during this time. Given that the data was exclusively generated from virtual behavioral medicine patients who had self-selected to receive care in this fashion across (often) multiple years, the findings may also not generalize across all healthcare populations; however, given the high prevalence of mental health disorders [48] and near-universal use of smartphones, these findings further develop the potential to assess smartphone usage and its relationship with symptoms of sleep disturbances, anxiety, and depression.

When quantifying behavior, it is important to consider the impact that the act of observation will have on its natural state. Indeed, it has been suggested that even passive tracking can influence smartphone usage [49]; however, given the extended duration of the data collection, which ranged from 15 days to 1088 days (Mean = 310, see Table 2), it is likely that this potential limitation was overcome.

Regarding our framework to assess inferred sleep, the ISP, ESP, and overlap percentage were established by defined measurements, whereas the nature of disruptions is not necessarily as clear. Specifically, disruptions may stem from awakening and subsequently using the smartphone, or from incoming communications to the smartphone leading to disruption. Further clarification of the nature of disruptions is important as those from alerts or external communication could be remedied by using sleep mode on the smartphone while usage upon awakening during the night would likely require education and counseling.

Another area that was not addressed in this study was the characterization of weekday-weekend differences in the behavioral measures, and how those differences may be related to mental health symptom status. The present study focused on 2-week aggregate behavioral measures as a representative value encompassing both weekday and weekend variation; however, a cursory post hoc analysis was conducted on the data (see Supplementary Material S1 Part 8) investigating the relationship between weekday-weekend differences across age groups for the presented behavioral metrics. The analysis identified a 2%–3% difference in weekday versus weekend overlap percentages across groups of 40 years and above. No consistent differences across weekday-weekend behaviors were observed for disruptions, but a general decreasing trend towards fewer disruptions in older age groups was present. Although there is limited data regarding objective measures of nocturnal smartphone usage parameters, these exploratory weekday-weekend results for disruptions are consistent with a previous study evaluating overall usage patterns across weekdays and weekends where no significant differences in usage patterns were found [50]. While the observed weekday-weekend differences in behaviors from our post hoc exploratory analysis were not large at the population level, the analysis did not include information on employment status, seasonal variations, or other critical measures necessary to fully contextualize such behavior; however, this study does highlight the potential value in the presented methodology to begin addressing such an important topic.

## Conclusions

In this study, we analyzed the associations between passively sensed human-smartphone interactions and clinical outcomes in a large clinical cohort, and we provide evidence that supports the utility of human-smartphone interaction behavior as an unobtrusive monitoring tool for symptoms that are highly prevalent in both clinical and nonclinical populations—specifically sleep disturbances, anxiety, and depression. This provides a basis for further study and refinement of the associations between human-smartphone behaviors, and their timing, with self-reported symptoms to integrate this model into clinical practice.

## Availability of Data and Materials

Public provisioning of Mindstrong healthcare data is not permissible per privacy policy and terms of service; however, individual requests for data may be made to the corresponding author and will be triaged to an appropriate privacy officer for consideration on a case-by-case basis. The modeling framework presented here has been filed with United States Patent Office as Provisional Application No. 63/442,010.

## Supplementary Material

zpad027_suppl_Supplementary_Materials

## Notes

Co-author review: All authors have seen and approved the final version of the manuscript.
