# Supplementary material for: Associations of smartphone usage patterns with sleep and mental health symptoms in a clinical cohort receiving virtual behavioral medicine care: a retrospective study
Source: Sleep Adv. 2023 Jul 5;4(1):zpad027. doi: 10.1093/sleepadvances/zpad027 (PMC10359037; doi:10.1093/sleepadvances/zpad027)
Supplement: zpad027_suppl_Supplementary_Materials [file zpad027_suppl_supplementary_materials.docx]

Supplementary Material for:

**Associations of Smartphone Usage Patterns with Sleep and Mental Health Symptoms in a Clinical Cohort Receiving Virtual Behavioral Medicine Care: A Retrospective Study**

Jonathan Knights^1a^, Jacob Shen^1a^, Vincent Mysliwiec^2^, Holly DuBois^1^

At time of submission: ^1^ Mindstrong Health, Menlo Park, California, USA, ^1a^ Department of Applied Science, ^2^ Department of Psychiatry and Behavioral Sciences, University of Texas Health Science Center at San Antonio, San Antonio, Texas

**Corresponding Authors**: Jonathan Knights, Department of Applied Science, SonderMind, 3000 Lawrence St, Denver, CO 80205, USA or [JonathanKnights.3783@gmail.com](mailto:jon.knights@mindstronghealth.com).

Phone Inactivity Analysis

[Part 1 Univariate Analysis for Demographics (Whole Sample) 4](#_Toc137113586)

[Depression 4](#_Toc137113587)

[Anxiety 4](#_Toc137113588)

[Sleep 4](#_Toc137113589)

[Part 2 Summary Statistics (For Multivariate Analysis) 5](#_Toc137113590)

[Table S2.1. Descriptives of Human-Smartphone Interaction Behaviors and Correlations with Sleep Disturbance and Mental Health Symptoms. 5](#_Toc137113591)

[Table S2.2. Summary for Number of Days during 14-Day Window 5](#_Toc137113592)

[Table S2.4: ANOVA for No. Days of smartphone with age group (excluded the age < 20 with only 1 patient): 5](#_Toc137113593)

[Part 3: Distributional Characterization and Assessment of Data Cleaning by Cook’s Distance 6](#_Toc137113594)

[Figure S3.1: Histograms of Behavior Measures, Sleep Disturbance, and Mental Health Symptoms from unadjusted data set (prior to removal of extreme Cook’s distance values). 6](#_Toc137113595)

[Table S3.1: Amount of observations removed from unadjusted data set identified by Cook’s distance 6](#_Toc137113596)

[Figure S3.2: Assessment of the impact of data cleaning by Cook’s distance on individual data 7](#_Toc137113597)

[Table S3.1: Data for removed “outliers” 7](#_Toc137113598)

[Figure S3.3: Distribution of behavioral measures after data cleaning with Cook’s distance 8](#_Toc137113599)

[Table S3.2: Comparison of symptom severity survey report between unadjusted and adjusted data sets 8](#_Toc137113600)

[Figure S4.4: Average survey count across symptom acuity spectrum 9](#_Toc137113601)

[Tables S3.3: Comparison of average survey count across symptom acuity spectrum 9](#_Toc137113602)

[Part 4 Multivariate Analysis for Behavioral Measures 13](#_Toc137113603)

[Depression 13](#_Toc137113604)

[Unadjusted Model 13](#_Toc137113605)

[Unadjusted Model (Remove outliers with cook’s distance) 13](#_Toc137113606)

[Adjusted Model (Remove outliers with cook’s distance) 13](#_Toc137113607)

[Anxiety 15](#_Toc137113608)

[Unadjusted Model 15](#_Toc137113609)

[Unadjusted Model (Remove outliers with cook’s distance) 15](#_Toc137113610)

[Adjusted Model (Remove outliers with cook’s distance) 15](#_Toc137113611)

[Sleep 17](#_Toc137113612)

[Unadjusted Model 17](#_Toc137113613)

[Unadjusted Model (Remove outliers with cook’s distance) 17](#_Toc137113614)

[Adjusted Model (Remove outliers with cook’s distance) 17](#_Toc137113615)

[Model Diagnostics 18](#_Toc137113616)

[Part 6 Test of Demographic Variables 20](#_Toc137113617)

[T-test to evaluate the mean difference between age and other demographic variables 20](#_Toc137113618)

[Fisher’s exact test to evaluate the association between two binary demographic variables 20](#_Toc137113619)

[Part 7 Empirical CDF Plots from Bootstrap 21](#_Toc137113620)

[Part 8: Assessment of weekday-weekend regularity and age in the analysis sample population 22](#_Toc137113621)

# Part 1 Univariate Analysis for Demographics (Whole Sample)

## Depression

|  | Estimate | Std. Error | Pr(>\|t\|) |
| --- | --- | --- | --- |
| age | -0.0048960 | 0.0017405 | 0.0049482 |
| femaleyes | -0.0233862 | 0.0448559 | 0.6021642 |
| locationurban | -0.0786272 | 0.0396346 | 0.0473956 |
| mood_bipolaryes | -0.0820367 | 0.0487876 | 0.0928045 |
| mood_depr_majoryes | 0.0764597 | 0.0399229 | 0.0555938 |
| personality_disorderyes | 0.3212795 | 0.1047963 | 0.0021992 |
| psychoses_schizoyes | -0.2823318 | 0.0788725 | 0.0003515 |

## Anxiety

|  | Estimate | Std. Error | Pr(>\|t\|) |
| --- | --- | --- | --- |
| age | -0.0132245 | 0.0017982 | 0.0000000 |
| femaleyes | 0.1015725 | 0.0468544 | 0.0302721 |
| locationurban | -0.0697545 | 0.0413892 | 0.0920583 |
| mood_bipolaryes | 0.0746164 | 0.0510239 | 0.1437749 |
| mood_depr_majoryes | -0.1149049 | 0.0417081 | 0.0059158 |
| personality_disorderyes | 0.3471263 | 0.1099614 | 0.0016173 |
| psychoses_schizoyes | -0.0805324 | 0.0826653 | 0.3300626 |

## Sleep

|  | Estimate | Std. Error | Pr(>\|t\|) |
| --- | --- | --- | --- |
| age | -0.0086211 | 0.0021123 | 0.0000463 |
| femaleyes | -0.0158460 | 0.0545512 | 0.7714764 |
| locationurban | -0.0722103 | 0.0481772 | 0.1340508 |
| mood_bipolaryes | 0.0441846 | 0.0592166 | 0.4556555 |
| mood_depr_majoryes | -0.0217430 | 0.0485401 | 0.6542402 |
| personality_disorderyes | 0.3227581 | 0.1268434 | 0.0110149 |
| psychoses_schizoyes | -0.3452648 | 0.0959326 | 0.0003265 |

# Part 2 Summary Statistics (For Multivariate Analysis)

## Table S2.1. Descriptives of Human-Smartphone Interaction Behaviors and Correlations with Sleep Disturbance and Mental Health Symptoms.

|  | Measure (Unit) | Mean (SD) |  | | | | |
| --- | --- | --- | --- | --- | --- | --- | --- |
|  |  |  | 1 | 2 | 3 | 4 | 5 |
| 1 | Overlap percentage (percentage) | 0.75 (0.18) |  |  |  |  |  |
| 2 | Disruption (fifteen-minute bin) | 4.85 (3.03) | -0.074 |  |  |  |  |
| 3 | Duration of the expected sleep period (hour) | 8.37 (2.26) | 0.60 | 0.13 |  |  |  |
| 4 | Self-Reported Sleep Disturbance | 2.16 (1.37) | -0.10 | 0.17 | -0.052 |  |  |
| 5 | Depressive Symptoms | 2.15 (1.13) | -0.039 | 0.11 | 0.027 | 0.47 |  |
| 6 | Anxiety Symptoms | 1.86 (1.12) | -0.028 | 0.11 | 0.033 | 0.44 | 0.66 |

##

## Table S2.2. Summary for Number of Days during 14-Day Window

|  | # days < 3 during 14-day window |
| --- | --- |
| Depression | 3.87% |
| Anxiety | 3.89% |
| Sleep | 3.9% |

## Table S2.4: ANOVA for No. Days of smartphone with age group (excluded the age < 20 with only 1 patient):

|  | Df | Sum Sq | Mean Sq | F value | Pr(>F) |
| --- | --- | --- | --- | --- | --- |
| age_cat | 5 | 682641.6 | 136528.32 | 3.547404 | 0.0033734 |
| Residuals | 2337 | 89943701.1 | 38486.82 | NA | NA |

# Part 3: Distributional Characterization and Assessment of Data Cleaning by Cook’s Distance

## Figure S3.1: Histograms of Behavior Measures, Sleep Disturbance, and Mental Health Symptoms from unadjusted data set (prior to removal of extreme Cook’s distance values).


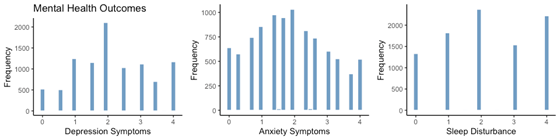

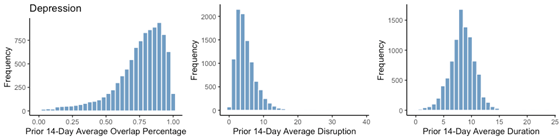

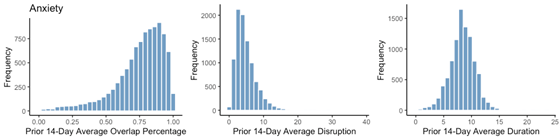

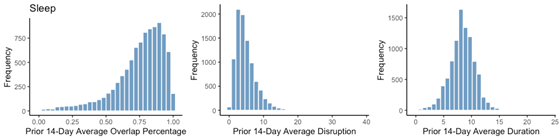


## Table S3.1: Amount of observations removed from unadjusted data set identified by Cook’s distance

|  | Population-level % removed |
| --- | --- |
| Depression | 5.89% |
| Anxiety | 5.70% |
| Sleep | 5.99% |

## Figure S3.2: Assessment of the impact of data cleaning by Cook’s distance on individual data


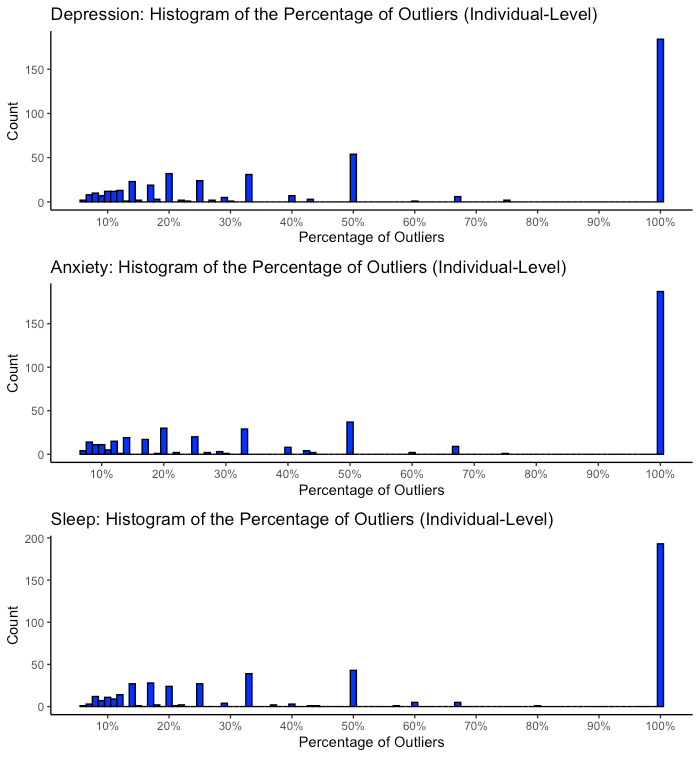


Figure 3.2 highlights that the majority of data removal was in fact identifying whole patient data sets that were disproportionately impactful to the regression analysis. Upon further inspection (See Table 3.1 and Figure 3.3 below) the data is predominately populated by higher-severity individuals with only 1-3 survey samples available, suggesting a potential subgroup of patients who may require further characterization and more focused future analysis. That being said, Table S3.1 below highlights that for the removed data points, a comparable fraction also came from low-level symptom patients as well, albeit not complete removal of the IDs

## Table S3.1: Data for removed “outliers”

| No. Unique Members | N (%)  Average Severity L0 | N (%)  Average Severity L1 | N (%)  Average Severity L2 | N (%)  Average Severity L3 | N (%)  Average Severity L4 |
| --- | --- | --- | --- | --- | --- |
| Depression | 116 (24.8%) | 43 (9.2%) | 68 (14.6%) | 46 (9.9%) | 194 (41.5%) |
| Anxiety | 120 (27.6%) | 46 (10.6%) | 58 (13.3%) | 98 (22.5%) | 113 (26.0%) |
| Sleep | 138 (29.6%) | 57 (12.2%) | 47 (10.1%) | 40 (8.57%) | 185 (39.6%) |

## Figure S3.3: Distribution of behavioral measures after data cleaning with Cook’s distance


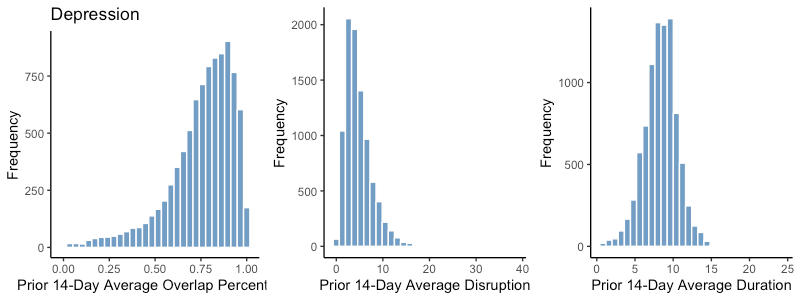


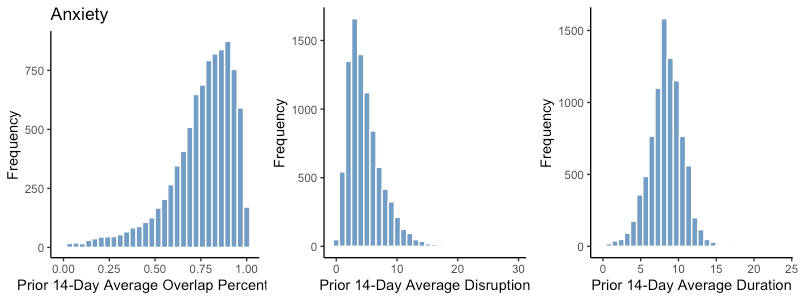


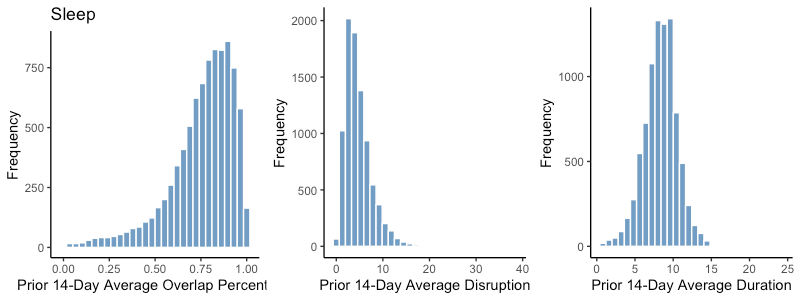


## Table S3.2: Comparison of symptom severity survey report between unadjusted and adjusted data sets

| KS test on the CDF of the distribution of average symptom severity in unadjusted and adjusted data sets | P-value from Kolmogorov-Smirnov Test (Aggregated by ID) | P-value from Kolmogorov-Smirnov Test (Population Comparison) |
| --- | --- | --- |
| Depression | 0.451 | 0.145 |
| Anxiety | 0.293 | 0.2267 |
| Sleep | 0.491 | 0.4175 |

## Figure S4.4: Average survey count across symptom acuity spectrum


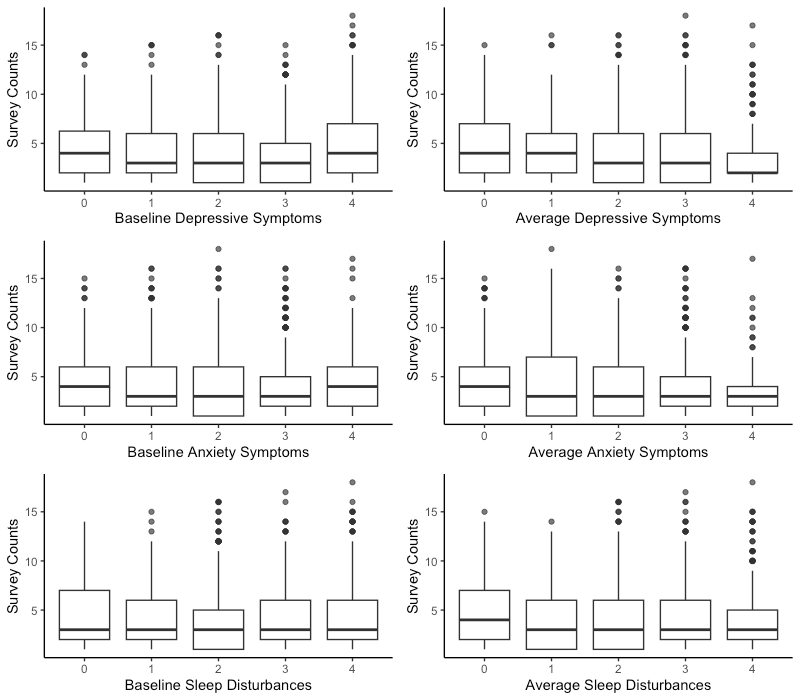


## Tables S3.3: Comparison of average survey count across symptom acuity spectrum

| **Depression**  **(survey count)** | **Mean (SD) (dep = 0), %ID** | **Mean (SD) (dep = 1), %ID** | **Mean (SD) (dep = 2), %ID** | **Mean (SD) (dep = 3), %ID** | **Mean (SD) (dep = 4), %ID** | **P-value (ANOVA)** | **P-value (Kruskal-Wallis H)** |
| --- | --- | --- | --- | --- | --- | --- | --- |
| **Baseline** | 4.62 (3.09), 6.7% | 4.22 (3.36), 10.2% | 3.93 (3.18), 37.0% | 3.74 (3.00), 27.1% | 4.97 (3.69), 19.0% | 1.4e-08*** | 1.158e-09*** |
| **Average** | 4.98 (3.30), 8.3% | 4.57 (3.38), 11.8% | 3.98 (3.14), 41.0% | 4.22 (3.58), 28.5% | 3.53 (2.93), 10.5% | 2.04e-05*** | 1.701e-06*** |

**Note that while the differences are “significant” the deltas across the different symptom domains are roughly 1-2 surveys, with significance being driven by the large sample size.**

TukeyHSD:

Baseline:

| Tukey multiple comparisons of means      95% family-wise confidence level  Fit: aov(formula = survey_count ~ bl_response_cat, data = dep_dat_agg)  $bl_response_cat            diff         lwr         upr     p adj  1-0 -0.4032805 -1.35348150  0.54692042 0.7749061  2-0 -0.6934932 -1.49643184  0.10944554 0.1274392  3-0 -0.8873509 -1.71244756 -0.06225432 0.0277436  4-0  0.3483656 -0.51028748  1.20701872 0.8026035  2-1 -0.2902126 -0.96418559  0.38376037 0.7654187  3-1 -0.4840704 -1.18429426  0.21615347 0.3244139  4-1  0.7516462  0.01217731  1.49111501 0.0441549  3-2 -0.1938578 -0.67566910  0.28795353 0.8074099  4-2  1.0418588  0.50460384  1.57911369 0.0000013  4-3  1.2357166  0.66587733  1.80555578 0.0000000 |
| --- |

Average:

| Tukey multiple comparisons of means      95% family-wise confidence level  Fit: aov(formula = survey_count ~ avg_response_cat, data = dep_dat_agg)  $avg_response_cat            diff        lwr          upr     p adj  1-0 -0.4090215 -1.2765684  0.458525501 0.6992140  2-0 -1.0080150 -1.7375466 -0.278483444 0.0015588  3-0 -0.7595773 -1.5155086 -0.003646077 0.0482240  4-0 -1.4525385 -2.3417358 -0.563341170 0.0000844  2-1 -0.5989935 -1.2306507  0.032663625 0.0727311  3-1 -0.3505559 -1.0125276  0.311415840 0.5980128  4-1 -1.0435170 -1.8543476 -0.232686370 0.0041190  3-2  0.2484377 -0.2182368  0.715112142 0.5930367  4-2 -0.4445235 -1.1056020  0.216555071 0.3531333  4-3 -0.6929611 -1.3830630 -0.002859238 0.0484712 |
| --- |

| **Anxiety**  **(survey count)** | **Mean (SD) (anx = 0), %ID** | **Mean (SD) (anx = 1), %ID** | **Mean (SD) (anx = 2), %ID** | **Mean (SD) (anx = 3), %ID** | **Mean (SD) (anx = 4), %ID** | **P-value (ANOVA)** | **P-value (Kruskal-Wallis H)** |
| --- | --- | --- | --- | --- | --- | --- | --- |
| **Baseline** | 4.40 (3.13), 15.2% | 4.19 (3.35), 19.9% | 3.96 (3.25), 33.7% | 4.03 (3.27), 23.4% | 4.57 (3.21), 7.8% | 0.104 | 0.001398*** |
| **Average** | 4.57 (3.14), 19.6% | 4.39 (3.68), 15.8% | 4.03 (3.22), 35.8% | 3.93 (3.20), 22.9% | 3.52 (2.69), 5.9% | 0.00219** | 0.000336*** |

TukeyHSD:

Baseline:

| Tukey multiple comparisons of means      95% family-wise confidence level  Fit: aov(formula = survey_count ~ bl_response_cat, data = anx_dat_agg)  $bl_response_cat             diff        lwr       upr     p adj  1-0 -0.20956945 -0.8634578 0.4443189 0.9061250  2-0 -0.43437107 -1.0278521 0.1591099 0.2671443  3-0 -0.36299094 -0.9956646 0.2696827 0.5192641  4-0  0.17193920 -0.6737931 1.0176715 0.9813346  2-1 -0.22480162 -0.7672266 0.3176233 0.7898681  3-1 -0.15342149 -0.7384695 0.4316265 0.9529038  4-1  0.38150865 -0.4292122 1.1922295 0.7007025  3-2  0.07138012 -0.4452734 0.5880336 0.9957038  4-2  0.60631026 -0.1565252 1.3691458 0.1915540  4-3  0.53493014 -0.2587791 1.3286393 0.3507369 |
| --- |

Average:

| Tukey multiple comparisons of means      95% family-wise confidence level  Fit: aov(formula = survey_count ~ avg_response_cat, data = anx_dat_agg)  $avg_response_cat            diff        lwr           upr     p adj  1-0 -0.1762519 -0.8237817  0.4712778908 0.9463006  2-0 -0.5382173 -1.0768216  0.0003869758 0.0502692  3-0 -0.6389522 -1.2284809 -0.0494234925 0.0259074  4-0 -1.0418229 -1.9424355 -0.1412103796 0.0139207  2-1 -0.3619654 -0.9401931  0.2162623687 0.4285174  3-1 -0.4627003 -1.0886371  0.1632365069 0.2575239  4-1 -0.8655710 -1.7904254  0.0592834301 0.0792798  3-2 -0.1007349 -0.6131767  0.4117068886 0.9835339  4-2 -0.5036056 -1.3557532  0.3485419299 0.4888399  4-3 -0.4028707 -1.2880854  0.4823439302 0.7263700 |
| --- |

| **Sleep**  **(survey count)** | **Mean (SD) (sleep = 0), %ID** | **Mean (SD) (sleep = 1), %ID** | **Mean (SD) (sleep = 2), %ID** | **Mean (SD) (sleep = 3), %ID** | **Mean (SD) (sleep = 4), %ID** | **P-value (ANOVA)** | **P-value (Kruskal-Wallis H)** |
| --- | --- | --- | --- | --- | --- | --- | --- |
| **Baseline** | 4.40 (3.07), 10.8% | 4.11 (3.07), 17.9% | 3.80 (3.27), 29.2% | 4.08 (3.28), 19.9% | 4.40 (3.36), 22.2% | 0.0234* | 0.0001137*** |
| **Average** | 4.83 (3.21), 13.5% | 3.95 (3.10), 14.8% | 3.90 (3.30), 29.9% | 4.32 (3.28), 23.3% | 3.78 (3.14), 18.4% | 8.16e-05*** | 9.567e-08*** |

TukeyHSD:

Baseline:

| Tukey multiple comparisons of means      95% family-wise confidence level  Fit: aov(formula = survey_count ~ bl_response_cat, data = sleep_dat_agg)  $bl_response_cat               diff         lwr        upr     p adj  1-0 -0.2921052632 -1.03029218 0.44608165 0.8167483  2-0 -0.5996779388 -1.28172164 0.08236576 0.1155263  3-0 -0.3198113208 -1.04341234 0.40378970 0.7475650  4-0 -0.0008492569 -0.71164002 0.70994151 1.0000000  2-1 -0.3075726757 -0.88306108 0.26791573 0.5892846  3-1 -0.0277060576 -0.65188690 0.59647479 0.9999514  4-1  0.2912560063 -0.31802785 0.90053986 0.6880960  3-2  0.2798666181 -0.27678895 0.83652219 0.6453653  4-2  0.5988286819  0.05893008 1.13872729 0.0209913  4-3  0.3189620639 -0.27256589 0.91049002 0.5807911 |
| --- |

Average:

| Tukey multiple comparisons of means      95% family-wise confidence level  Fit: aov(formula = survey_count ~ avg_response_cat, data = sleep_dat_agg)  $avg_response_cat             diff         lwr         upr     p adj  1-0 -0.88323655 -1.60212987 -0.16434322 0.0072337  2-0 -0.93304188 -1.55950250 -0.30658126 0.0004752  3-0 -0.50467152 -1.15804503  0.14870199 0.2165857  4-0 -1.04610503 -1.73051028 -0.36169977 0.0003019  2-1 -0.04980533 -0.65677761  0.55716694 0.9994421  3-1  0.37856503 -0.25614702  1.01327708 0.4792956  4-1 -0.16286848 -0.82948155  0.50374459 0.9634136  3-2  0.42837036 -0.09936507  0.95610578 0.1740436  4-2 -0.11306315 -0.67876474  0.45263844 0.9824827  4-3 -0.54143351 -1.13680090  0.05393388 0.0948839 |
| --- |

# Part 4 Multivariate Analysis for Behavioral Measures

## Depression

### Unadjusted Model

|  | Estimate | 95% CI | P-value |
| --- | --- | --- | --- |
| (Intercept) | 2.1697 | (2.1312,2.2082) | 0 |
| wp.mean_overlap_percentage | -0.1042 | (-0.2716,0.0631) | 0.2224 |
| wp.mean_disruption | -8e-04 | (-0.0125,0.011) | 0.8981 |
| wp.mean_duration | 0.0211 | (0.0037,0.0386) | 0.0178 |
| bp.mean_overlap_percentage | -0.7113 | (-1.0374,-0.3852) | 0 |
| bp.mean_disruption | 0.0508 | (0.0364,0.0653) | 0 |
| bp.mean_duration | 0.0532 | (0.0276,0.0787) | 0 |
| variance_random_(Intercept) | 0.6871 |  |  |
| variance_random_wp.mean_overlap_percentage | 0.1936 |  |  |
| variance_random_wp.mean_disruption | 0.0042 |  |  |
| variance_random_wp.mean_duration | 0.0093 |  |  |
| variance_random_residual | 0.5385 |  |  |

### Unadjusted Model (Remove outliers with cook’s distance)

|  | Estimate | 95% CI | P-value |
| --- | --- | --- | --- |
| (Intercept) | 2.1618 | (2.1234,2.2002) | 0 |
| wp.mean_overlap_percentage | 0.0153 | (-0.1389,0.1695) | 0.8459 |
| wp.mean_disruption | 2e-04 | (-0.0103,0.0107) | 0.9716 |
| wp.mean_duration | 0.0055 | (-0.0103,0.0212) | 0.4986 |
| bp.mean_overlap_percentage | -0.722 | (-1.0641,-0.38) | 0 |
| bp.mean_disruption | 0.057 | (0.0419,0.0722) | 0 |
| bp.mean_duration | 0.0529 | (0.0267,0.0791) | 1e-04 |
| variance_random_(Intercept) | 0.6616 |  |  |
| variance_random_wp.mean_overlap_percentage | 0.0201 |  |  |
| variance_random_wp.mean_disruption | 5e-04 |  |  |
| variance_random_wp.mean_duration | 0.0033 |  |  |
| variance_random_residual | 0.4325 |  |  |

### Adjusted Model (Remove outliers with cook’s distance)

Add demographic variables with p-value < 0.05 from univariate analysis

|  | Estimate | 95% CI Width | P-value |
| --- | --- | --- | --- |
| **(Intercept)** | **2.3655** | **(2.1476,2.5834)** | **0** |
| wp.mean_overlap_percentage | 0.0152 | (-0.1392,0.1696) | 0.847 |
| wp.mean_disruption | 4e-04 | (-0.0101,0.0109) | 0.9403 |
| wp.mean_duration | 0.0055 | (-0.0103,0.0212) | 0.4984 |
| **bp.mean_overlap_percentage** | **-0.7951** | **(-1.138,-0.4522)** | **0** |
| **bp.mean_disruption** | **0.0526** | **(0.0372,0.068)** | **0** |
| **bp.mean_duration** | **0.0607** | **(0.0341,0.0873)** | **0** |
| age | -0.0034 | (-0.0069,2e-04) | 0.0671 |
| femaleyes | -0.0202 | (-0.1082,0.0679) | 0.6538 |
| locationurban | -0.0646 | (-0.142,0.0129) | 0.1024 |
| **mood_depr_majoryes** | **0.0855** | **(0.0042,0.1668)** | **0.0395** |
| **personality_disorderyes** | **0.2795** | **(0.0749,0.484)** | **0.0075** |
| **psychoses_schizoyes** | **-0.2706** | **(-0.4325,-0.1088)** | **0.0011** |
| variance_random_(Intercept) | 0.6504 |  |  |
| variance_random_wp.mean_overlap_percentage | 0.0198 |  |  |
| variance_random_wp.mean_disruption | 5e-04 |  |  |
| variance_random_wp.mean_duration | 0.0033 |  |  |
| variance_random_residual | 0.4325 |  |  |

## Anxiety

### Unadjusted Model

|  | Estimate | 95% CI | P-value |
| --- | --- | --- | --- |
| (Intercept) | 1.9067 | (1.8664,1.947) | 0 |
| wp.mean_overlap_percentage | -0.1452 | (-0.306,0.0155) | 0.077 |
| wp.mean_disruption | 0.0087 | (-0.0023,0.0198) | 0.1211 |
| wp.mean_duration | -0.0026 | (-0.0185,0.0133) | 0.7477 |
| bp.mean_overlap_percentage | -0.5518 | (-0.8912,-0.2124) | 0.0015 |
| bp.mean_disruption | 0.0486 | (0.0337,0.0636) | 0 |
| bp.mean_duration | 0.0489 | (0.0224,0.0755) | 3e-04 |
| variance_random_(Intercept) | 0.8 |  |  |
| variance_random_wp.mean_overlap_percentage | 0.6678 |  |  |
| variance_random_wp.mean_disruption | 0.0047 |  |  |
| variance_random_wp.mean_duration | 0.0084 |  |  |
| variance_random_residual | 0.4337 |  |  |

### Unadjusted Model (Remove outliers with cook’s distance)

|  | Estimate | 95% CI | P-value |
| --- | --- | --- | --- |
| (Intercept) | 1.8856 | (1.845,1.9261) | 0 |
| wp.mean_overlap_percentage | -0.0704 | (-0.2104,0.0697) | 0.3248 |
| wp.mean_disruption | 0.0132 | (0.0036,0.0228) | 0.0071 |
| wp.mean_duration | -0.0077 | (-0.0218,0.0065) | 0.287 |
| bp.mean_overlap_percentage | -0.5605 | (-0.9201,-0.2009) | 0.0023 |
| bp.mean_disruption | 0.0584 | (0.0424,0.0744) | 0 |
| bp.mean_duration | 0.0552 | (0.0273,0.0831) | 1e-04 |
| variance_random_(Intercept) | 0.7718 |  |  |
| variance_random_wp.mean_overlap_percentage | 0.0021 |  |  |
| variance_random_wp.mean_disruption | 9e-04 |  |  |
| variance_random_wp.mean_duration | 0.0025 |  |  |
| variance_random_residual | 0.3525 |  |  |

### Adjusted Model (Remove outliers with cook’s distance)

Add demographic variables with p-value < 0.05 from univariate analysis

|  | Estimate | 95% CI Width | P-value |
| --- | --- | --- | --- |
| **(Intercept)** | **2.5083** | **(2.2798,2.7368)** | **0** |
| wp.mean_overlap_percentage | -0.0706 | (-0.2108,0.0696) | 0.3238 |
| **wp.mean_disruption** | **0.0134** | **(0.0038,0.023)** | **0.0063** |
| wp.mean_duration | -0.0078 | (-0.0219,0.0063) | 0.2801 |
| **bp.mean_overlap_percentage** | **-0.6613** | **(-1.0201,-0.3024)** | **3e-04** |
| **bp.mean_disruption** | **0.0478** | **(0.0315,0.064)** | **0** |
| **bp.mean_duration** | **0.0685** | **(0.0404,0.0966)** | **0** |
| **age** | **-0.0108** | **(-0.0145,-0.007)** | **0** |
| femaleyes | 0.0768 | (-0.0159,0.1695) | 0.1045 |
| locationurban | -0.0759 | (-0.157,0.0053) | 0.067 |
| mood_depr_majoryes | -0.0777 | (-0.1631,0.0078) | 0.0749 |
| personality_disorderyes | 0.2116 | (-0.0067,0.4298) | 0.0576 |
| **psychoses_schizoyes** | **-0.1745** | **(-0.343,-0.0061)** | **0.0424** |
| variance_random_(Intercept) | 0.7518 |  |  |
| variance_random_wp.mean_overlap_percentage | 0 |  |  |
| variance_random_wp.mean_disruption | 8e-04 |  |  |
| variance_random_wp.mean_duration | 0.0023 |  |  |
| variance_random_residual | 0.3527 |  |  |

## Sleep

### Unadjusted Model

|  | Estimate | 95% CI | P-value |
| --- | --- | --- | --- |
| (Intercept) | 2.1722 | (2.1258,2.2186) | 0 |
| wp.mean_overlap_percentage | -0.244 | (-0.4624,-0.0257) | 0.0289 |
| wp.mean_disruption | 0.036 | (0.0214,0.0506) | 0 |
| wp.mean_duration | -0.0156 | (-0.0378,0.0067) | 0.1706 |
| bp.mean_overlap_percentage | -0.7874 | (-1.1801,-0.3947) | 1e-04 |
| bp.mean_disruption | 0.0798 | (0.0625,0.0971) | 0 |
| bp.mean_duration | 0.0087 | (-0.0221,0.0394) | 0.5799 |
| variance_random_(Intercept) | 0.9644 |  |  |
| variance_random_wp.mean_overlap_percentage | 1.0457 |  |  |
| variance_random_wp.mean_disruption | 0.0051 |  |  |
| variance_random_wp.mean_duration | 0.0175 |  |  |
| variance_random_residual | 0.8202 |  |  |

### Unadjusted Model (Remove outliers with cook’s distance)

|  | Estimate | 95% CI | P-value |
| --- | --- | --- | --- |
| (Intercept) | 2.1881 | (2.1407,2.2356) | 0 |
| wp.mean_overlap_percentage | -0.2101 | (-0.4074,-0.0128) | 0.037 |
| wp.mean_disruption | 0.0452 | (0.0319,0.0585) | 0 |
| wp.mean_duration | -0.03 | (-0.05,-0.0101) | 0.0033 |
| bp.mean_overlap_percentage | -0.8265 | (-1.2524,-0.4007) | 1e-04 |
| bp.mean_disruption | 0.1013 | (0.0827,0.12) | 0 |
| bp.mean_duration | 0.0049 | (-0.0275,0.0374) | 0.766 |
| variance_random_(Intercept) | 0.9789 |  |  |
| variance_random_wp.mean_overlap_percentage | 0.2723 |  |  |
| variance_random_wp.mean_disruption | 0.0012 |  |  |
| variance_random_wp.mean_duration | 0.0081 |  |  |
| variance_random_residual | 0.666 |  |  |

### Adjusted Model (Remove outliers with cook’s distance)

Add demographic variables with p-value < 0.05 from univariate analysis

|  | Estimate | 95% CI Width | P-value |
| --- | --- | --- | --- |
| **(Intercept)** | **2.4924** | **(2.2224,2.7624)** | **0** |
| **wp.mean_overlap_percentage** | **-0.2137** | **(-0.4112,-0.0161)** | **0.0342** |
| **wp.mean_disruption** | **0.0454** | **(0.0321,0.0588)** | **0** |
| **wp.mean_duration** | **-0.0302** | **(-0.0501,-0.0102)** | **0.0032** |
| **bp.mean_overlap_percentage** | **-0.9037** | **(-1.332,-0.4754)** | **0** |
| **bp.mean_disruption** | **0.0977** | **(0.0787,0.1167)** | **0** |
| bp.mean_duration | 0.015 | (-0.0179,0.048) | 0.3716 |
| age | -0.0044 | (-0.0089,0) | 0.0509 |
| femaleyes | -0.0012 | (-0.1104,0.1079) | 0.9824 |
| locationurban | -0.0654 | (-0.1611,0.0304) | 0.1809 |
| mood_depr_majoryes | -0.0052 | (-0.1059,0.0954) | 0.919 |
| personality_disorderyes | 0.1057 | (-0.1516,0.363) | 0.421 |
| **psychoses_schizoyes** | **-0.4019** | **(-0.6024,-0.2014)** | **1e-04** |
| variance_random_(Intercept) | 0.9709 |  |  |
| variance_random_wp.mean_overlap_percentage | 0.2771 |  |  |
| variance_random_wp.mean_disruption | 0.0012 |  |  |
| variance_random_wp.mean_duration | 0.0082 |  |  |
| variance_random_residual | 0.666 |  |  |
|  |  |  |  |

## Model Diagnostics


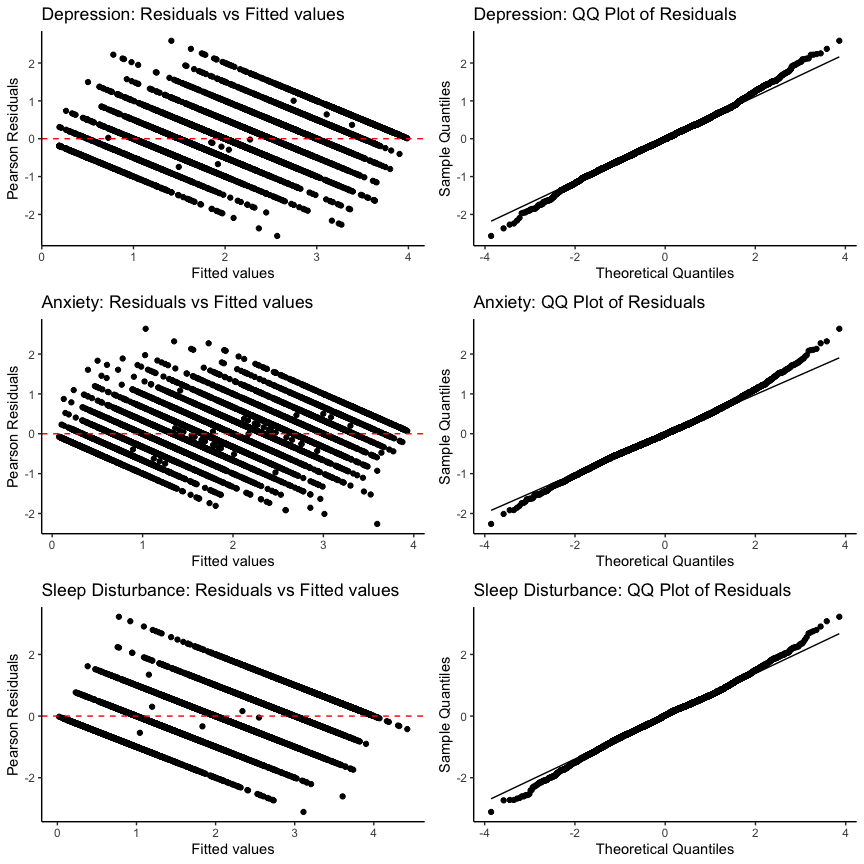


**Part 5 Boxplots of the between-person differences of overlap percentage or daily disruption and their corresponding depressive symptoms, anxiety symptoms or self-reported sleep disturbance.**

**
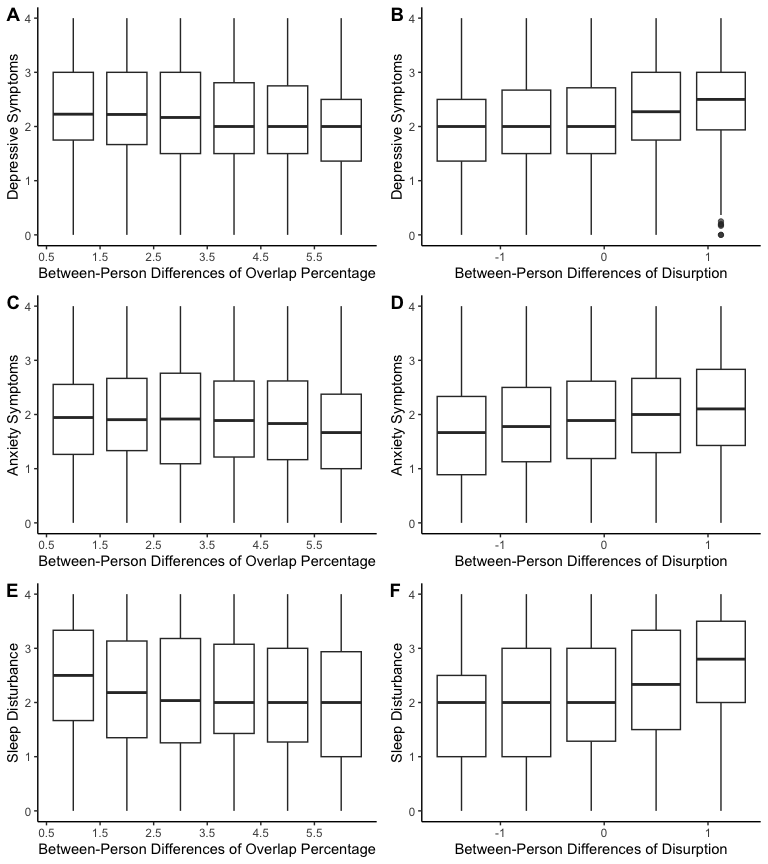
**

Panels A and B visualize the between-person associations between reported depressive symptom severity (y-axis) and overlap percentage and disruption, respectively (x-axis). Panels C and D visualize the between-person associations between reported anxiety symptom severity (y-axis) and overlap percentage and disruption, respectively (x-axis). Finally, panels E and F visualize the between-person associations between reported sleep disturbances (y-axis) and overlap percentage and disruption, respectively (x-axis). In all panels, the sample populations were equally binned into the number of groups visualized.

# Part 6 Test of Demographic Variables

## T-test to evaluate the mean difference between age and other demographic variables

|  | Mean Age 1 | Mean Age 2 | P-value (t-test) |
| --- | --- | --- | --- |
| Gender (1 - female, 2 - male) | 57.11 | 55.57 | 0.0047 |
| mood_depr_major (1 - yes, 2 - no) | 58.28 | 55.63 | 0.0000 |
| personality_disorder (1 - yes, 2 - no) | 50.90 | 56.92 | 0.0000 |
| psychoses_schizo (1 - yes, 2 - no) | 51.77 | 57.05 | 0.0000 |

## Fisher’s exact test to evaluate the association between two binary demographic variables

|  | female | mood_depr_major | personality_disorder | psychoses_schizo |
| --- | --- | --- | --- | --- |
| female | - | P-value: 1 | P-value: 0.0198 | P-value: 1e-04 |
| mood_depr_major | - | - | P-value: 0 | P-value: 0 |
| personality_disorder | - | - | - | P-value: 0.009 |
| psychoses_schizo | - | - | - | - |

# Part 7 Empirical CDF Plots from Bootstrap

Bootstrap method has been applied with 500 random samples using 20% of the original data per random sample, stratified by ID, e.g. each bootstrapped sample contains a random selection of 20% of the population and all associated data with that 20% (not a sampling of 20% of the observations).


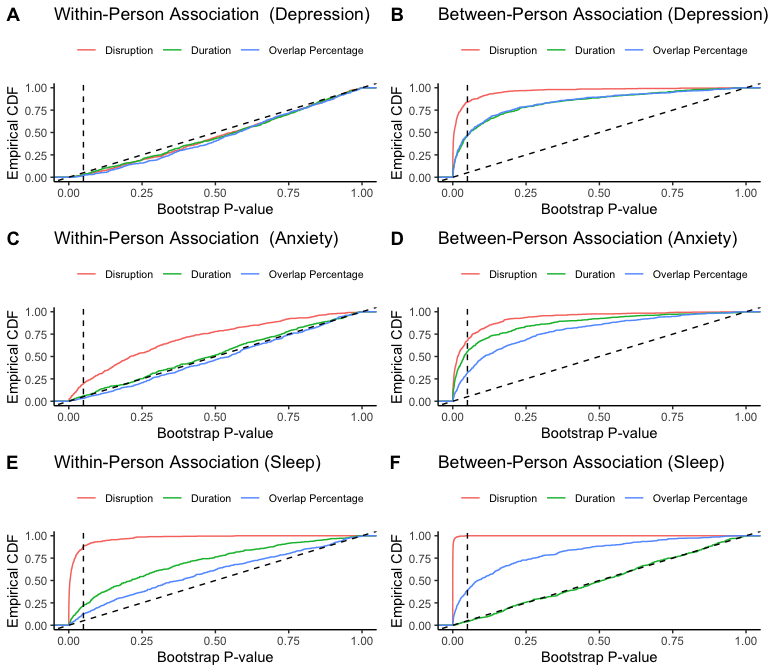


| **A** | Power | **B** | Power | **C** | Power |
| --- | --- | --- | --- | --- | --- |
| Overlap Percentage | - | Overlap Percentage | 46.8% | Overlap Percentage | - |
| Disruption | - | Disruption | 83.8% | Disruption | 19.4% |
| Duration | - | Duration | 47% | Duration | - |
| **D** | Power | **E** | Power | **F** | Power |
| Overlap Percentage | 31.8% | Overlap Percentage | 12.4% | Overlap Percentage | 38.8% |
| Disruption | 67.8% | Disruption | 88.2% | Disruption | 100% |
| Duration | 55.6% | Duration | 22% | Duration | - |

# Part 8: Assessment of weekday-weekend regularity and age in the analysis sample population

The following analysis represents a population level exploratory view of the differences in weekday versus weekend behaviors across age groups. All weekday and weekend observations for all patients were pooled into different groups and represent below. Non-parametric tests were conducted on the distributions given the skewed nature, and significance thresholds were adjusted using a conservative Bonferroni correction.


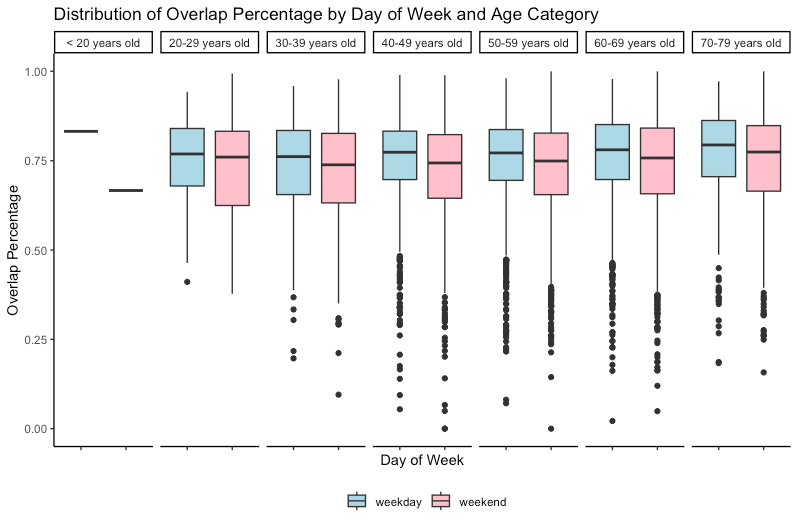


| **Overlap Percentage** | **Median (SD) - weekday** | **Median (SD) - weekend** | **P-value (Kolmogorov-Smirnov Test)** |
| --- | --- | --- | --- |
| **< 20 (N = 1)** | 0.832 (NA) | 0.667 (NA) | NA |
| **20 – 29 (N = 72)** | 0.769 (0.118) | 0.760 (0.141) | 0.631 |
| **30 – 39 (N = 290)** | 0.761 (0.140) | 0.738 (0.157) | 0.134 |
| **40 – 49 (N = 627)** | 0.774 (0.144) | 0.744 (0.156) | 0.0000571*** |
| **50 – 59 (N = 1,137)** | 0.772 (0.133) | 0.749 (0.144) | 0.0000118*** |
| **60 – 69 (N = 1,299)** | 0.780 (0.133) | 0.758 (0.152) | 0.0000477*** |
| **70 –79 (N = 480)** | 0.794 (0.131) | 0.774 (0.152) | 0.0425 |
| Multiple test significance threshold of *p*=0.008 set using Bonferroni correction for six tests on *p*=0.05 base threshold | | | |


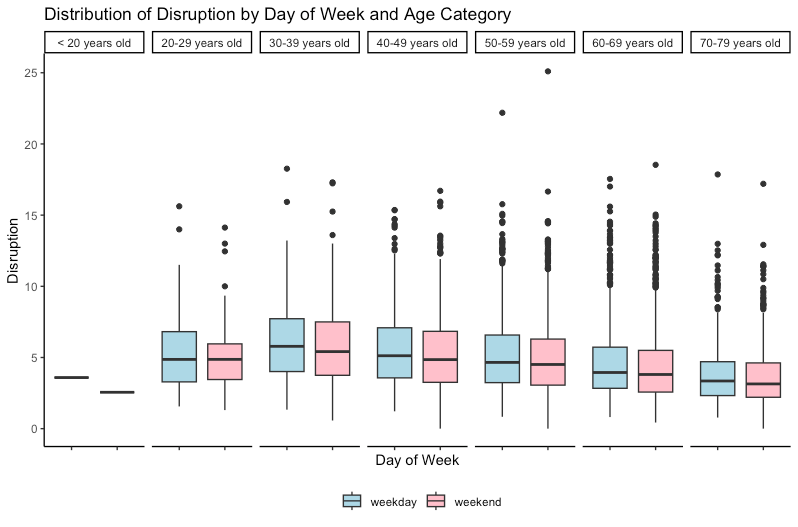


| **Disruption** | **Median (SD) - weekday** | **Median (SD) - weekend** | **P-value (Kolmogorov-Smirnov Test)** |
| --- | --- | --- | --- |
| **< 20 (N = 1)** | 3.59 (NA) | 2.56 (NA) | NA |
| **20 – 29 (N = 72)** | 4.86 (2.73) | 4.87 (2.55) | 0.768 |
| **30 – 39 (N = 290)** | 5.78 (2.73) | 5.41 (2.78) | 0.562 |
| **40 – 49 (N = 627)** | 5.12 (2.63) | 4.85 (2.72) | 0.0912 |
| **50 – 59 (N = 1,137)** | 4.65 (2.60) | 4.51 (2.57) | 0.0758 |
| **60 – 69 (N = 1,299)** | 3.95 (2.46) | 3.81 (2.48) | 0.00819 |
| **70 –79 (N = 480)** | 3.35 (2.23) | 3.14 (2.25) | 0.0987 |
| Multiple test significance threshold of *p*=0.008 set using Bonferroni correction for six tests on *p*=0.05 base threshold | | | |
